# DELTA: A Distal Enhancer Locating Tool Based on AdaBoost Algorithm and Shape Features of Chromatin Modifications

**DOI:** 10.1371/journal.pone.0130622

**Published:** 2015-06-19

**Authors:** Yiming Lu, Wubin Qu, Guangyu Shan, Chenggang Zhang

**Affiliations:** Beijing Institute of Radiation Medicine, State Key Laboratory of Proteomics, Beijing, 100850, PR China; Università degli Studi di Milano, ITALY

## Abstract

Accurate identification of DNA regulatory elements becomes an urgent need in the post-genomic era. Recent genome-wide chromatin states mapping efforts revealed that DNA elements are associated with characteristic chromatin modification signatures, based on which several approaches have been developed to predict transcriptional enhancers. However, their practical application is limited by incomplete extraction of chromatin features and model inconsistency for predicting enhancers across different cell types. To address these issues, we define a set of non-redundant shape features of histone modifications, which shows high consistency across cell types and can greatly reduce the dimensionality of feature vectors. Integrating shape features with a machine-learning algorithm AdaBoost, we developed an enhancer predicting method, DELTA (Distal Enhancer Locating Tool based on AdaBoost). We show that DELTA significantly outperforms current enhancer prediction methods in prediction accuracy on different datasets and can predict enhancers in one cell type using models trained in other cell types without loss of accuracy. Overall, our study presents a novel framework for accurately identifying enhancers from epigenetic data across multiple cell types.

## Background

Temporal and tissue-specific regulation of gene transcription requires cooperativeness of diverse *cis*-regulatory elements, such as transcriptional promoters, enhancers and insulators [1,2]. Transcriptional enhancers play a critical role in the regulation of tissue-specific gene expression and embody a large set of non-coding SNPs that may be linked to human disease phenotypes [3]. However, enhancers are not located at fixed distances from the genes they regulate, hence their identification is not trivial [1,4,5]. Early computational methods for predicting enhancers heavily relied on genome sequence context, and their performances were severely limited by the incomplete knowledge of transcription factor binding motifs and the inability to cope with condition-dependent data [6–10]. Experimental approaches were recently developed to identify enhancers by investigating the binding sites of transcriptional co-activators p300 and CBP [11], as they are known to be associated with functional enhancers [12,13]. Since not all enhancers are marked by a given set of co-activators, this approach could only identify a subset of enhancers [14,15].

Recently, it was shown that different elements, such as promoters and enhancers, were marked by distinct patterns of chromatin modifications. Specifically, Heintzman *et al*. found that promoters showed an enrichment of H3K4me3, H3ac and H4ac and a depletion of H3K4me1 and enhancers were enriched with H3K4me1, H3K4me2, H3ac and H4ac but lack of H3K4me3 [15]. In addition, emerging evidence suggested that different regulatory elements also exhibit different distributional patterns of modifications, such as broad or narrow, unimodal or bimodal, symmetric or asymmetric. Genome-wide mapping of multiple histone modifications in CD4^+^ T and other cell types showed that H3K4me2 and H3K4me3 at transcription start sites (TSSs) followed a bimodal distribution asymmetrically skewing towards the downstream coding region while their distribution around enhancers is unimodal and symmetric [15–17].

Supervised and unsupervised computational approaches were developed for identifying enhancers on the basis of chromatin modification mapping data. The supervised approaches used chromatin signatures surrounding known enhancers to predict new enhancers. Existing supervised approaches include profiling method (PM) [15], artificial neural networks (CSI-ANN) [18], hidden Markov model (Chromatin and Chromia) [7,19], support vector machine (ChromaGenSVM) [20] and random forests (RFECS) [21]. Some of them employed feature selection strategies such as genetic algorithm optimization [20] or out-of-bag method [21] to try to obtain an optimal subset of modifications for enhancer prediction. Unsupervised approaches were also developed, including ChromaSig [22], ChromHMM [23], ChAT [24] and Segway [25], to detect different chromatin states from the combinatorial patterns of chromatin modifications.

However, the practical application of these methods is limited by two primary constraints. First, feature extraction of chromatin signatures is usually lacking. Most previous methods predict enhancers only using the intensity of histone modification, which refers to the total number of sequencing reads or the maximum coverage depth in a genomic region. However, the distributions of chromatin modifications around regulatory elements are sequential profiles with distinct shape features. Constructing intensity vectors by dividing genomic regions into small bins, namely the “binned-vector” method, is a straightforward way to keep the distributional information of histone modifications around regulatory elements [21]. However, the consequent high-dimensional feature set would easily exceed the capabilities of machine learning algorithms and reduces the model interpretability, especially when the number of modifications is large. Therefore, an efficient dimensionality reduction method is still lacking. Second, model consistency across various cell types is not considered in previous methods. Most studies trained models and made predictions in the same cell type and failed to validate their methods on independent datasets, making them more vulnerable to the overfitting problem. Recent study has shown that models trained in one cell type made less accurate predictions in another cell type [21]. This problem will severely limit their application to enhancer prediction in new cell types.

To address these issues, here we present a novel method for enhancer prediction based on AdaBoost algorithm [26] and the shape features of chromatin modifications, showing that our model not only significantly outperforms previous methods in prediction accuracy but also exhibits an outstanding cell type generality during model training and prediction on diverse cell types.

## Results

### Quantification of the shape features of chromatin modifications at promoters and enhancers

**Fig 1:**
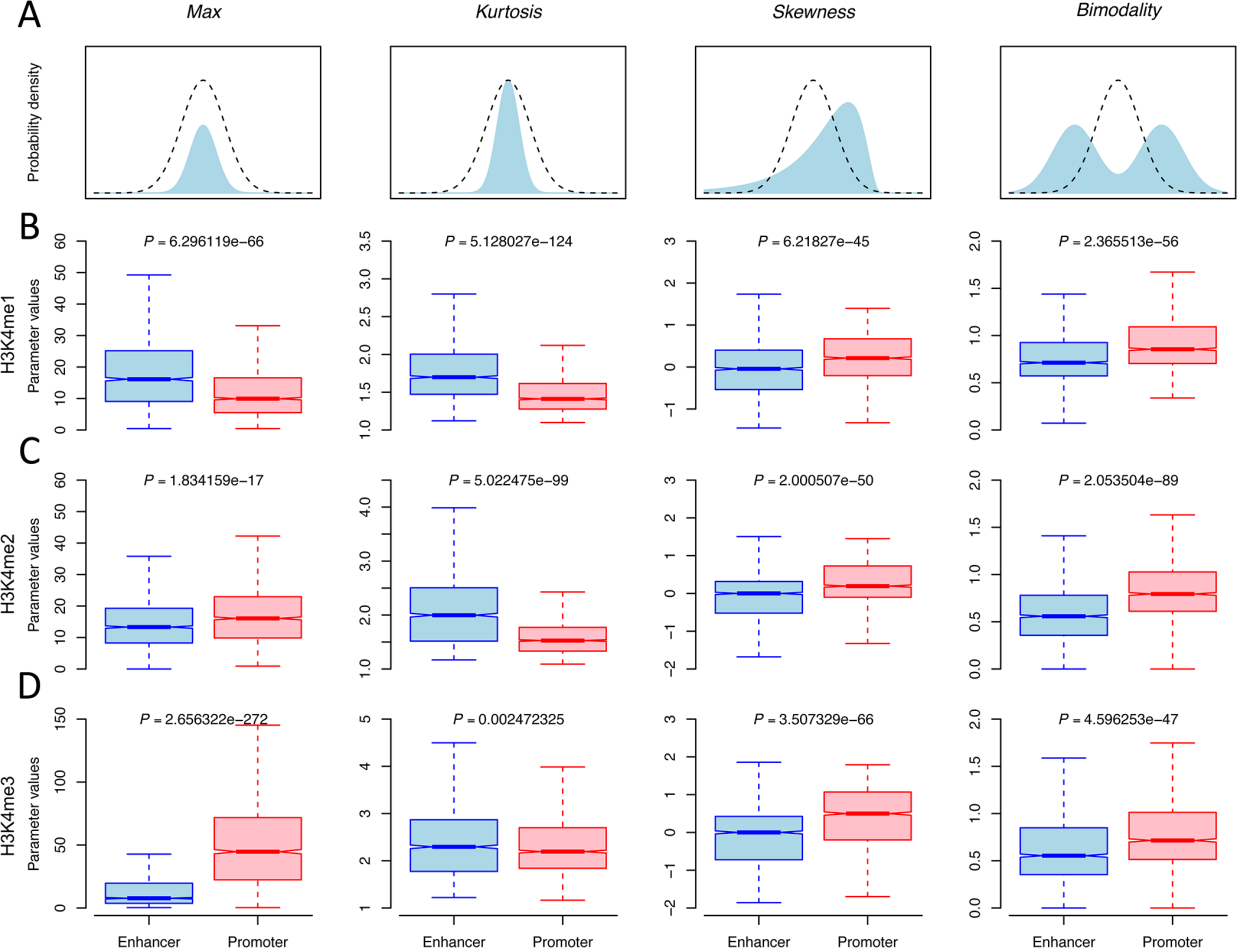
Quantification of intensity and shape features of ChIP-seq signals. A) Graphical representation of four parameters *Max*, *Kurtosis*, *Skewness* and *Bimodality*. The dashed curves indicate standard normal distribution, and the light blue area under curve indicates a probability distribution with the corresponding parameter changed. The boxplots of the four parameters and significances of the difference between enhancers and promoters for B) H3K4me1, C) H3K4me2 and D) H3K4me3 in CD4^+^ T cells. Significance (*P*-value) of the difference between two means was calculated by Wilcoxon rank-sum difference test.

We know from the technical principles of ChIP-seq that the number of sequencing reads mapped to a genomic region is proportional to the density of protein binding sites or chromatin modifications [27]. Therefore, the distribution of reads across a genomic region can be treated as a probability distribution reflecting the biological signals of chromatin modifications, which enables us to quantify the distributional features of chromatin modifications in a statistical way. Actually, descriptive parameters have been widely used to quantify the probability distribution of variables in probability theory and statistics. Here we introduce three parameters that are used in probability theory for describing the probability distribution of variables [28], including *Kurtosis*, *Skewness* and *Bimodality*, to quantify the shape features of chromatin modification profiles surrounding regulatory elements. Specifically, *Kurtosis* is introduced to quantify the “sharpness” of a ChIP-seq signal, *Skewness* quantifies the extent of a ChIP-seq signal “leans” to one side of the mean, and *Bimodality* is a parameter that quantifies a bimodally distributed ChIP-seq signal, as illustrated in Fig 1A. In addition, an intensity parameter *Max* is also introduced to describe the strength of a ChIP-seq signal. The definitions and calculation methods are described in detail in Materials and Methods.

Because enhancers and promoters are known to have distinct chromatin signatures, we first examined whether the defined parameters could accurately describe the characteristic patterns of histone modifications surrounding the two types of elements. We used the datasets of 38 and 26 histone modifications in CD4^+^ T and H1 cells to investigate the shape and intensity parameters at enhancers and promoters. p300 binding sites overlapping with DNase-I hypersensitive sites (DHSs) and distal (>2.5 kb) to UCSC TSSs are treated as representative of active enhancers, and UCSC TSSs overlapped with DHSs are treated as representative of active promoters. A total of 1983 and 6693 active and distal p300 sites and 9476 and 10663 active TSSs were included in this survey in CD4^+^ T and H1 cells, respectively.

We found that the shape parameters accurately quantify the known chromatin signatures of promoter and enhancer. The skewed distribution of histone modifications is an important feature of promoters, and several histone modifications enriched at promoters were reported to be skewed towards the downstream (transcribed region) of TSS [15,16]. We found the average *Skewness* of H3K4me1, H3K4me2, H3K4me3 and H3K9ac at promoters is negative, indicating their distribution is asymmetrically biased to the left (i.e., downstream) of TSS. In contrast, their average *Skewness* at enhancers is very close to zero, indicating their unbiased distribution at enhancers (S1 and S2 Tables). Another important feature of promoters is the bimodal distribution of histone modifications, implying depletion of nucleosomes at this position [7,15,29]. We found that the average *Bimodality* of H3K4me1, H3K4me2 and H3K4me3 at promoters is significantly larger than 5/9 (a value for unimodal distribution), indicating their distribution is generally bimodal (Fig 1B–1D). Their average *Bimodality* at enhancers is around 5/9, indicating their unimodal distribution at enhancers. We also noticed that most histone modifications at enhancers had larger *Kurtosis* than those at promoters, indicating the distributions of histone modifications at enhancers are sharper (or narrower) than those at promoters. In addition, we found that a subset of histone modifications displayed very distinct shape and intensity parameters between enhancers and promoters. Among all modifications, H3K4me1, H3K4me2 and H3K4me3 are three modifications exhibiting the most significant differences, as shown in Fig 1B–1D, and for two (H3K4me1 and H3K4me2) of them, shape parameters exhibit even more significant difference than *Max* does, suggesting the potential capability of shape features of histone modification in enhancer prediction.

We also investigated whether p300-positive and p300-negative enhancers have different histone modification profiles. We examined the shape and intensity parameters at a p300-independent transcriptional coactivator MED1 (TRAP220), which has been shown to occupy enhancers as well as promoters [12] in ENCODE regions, and compared them to those of distal p300 sites in HeLa cells. As shown in S1 Fig., all four parameters (*Kurtosis*, *Skewness*, *Bimodality* and *Max*) showed good consistency, and no significant difference were found between p300 and MED1-binding sites (*P* > 0.01, Wilcoxon test) for all three important modifications H3K4me1, H3K4me2 and H3K4me3, indicating p300-positive/negative enhancers share similar histone modification profiles.

### An AdaBoost-based framework for enhancer prediction

AdaBoost, short for “Adaptive Boosting”, is a machine learning meta-algorithm [30]. The core principle of AdaBoost is to fit a sequence of weak classifiers (in this case small decision trees) on repeatedly modified versions of the data to produce a powerful “committee”. Compared to bagging and random forests, AdaBoost adopts a cleverer way of averaging trees by increasing the weight of misclassified data at each iteration, hence can dramatically improve the accuracy of predictions [31,32]. Using the four extracted parameters *Kurtosis*, *Skewness*, *Bimodality* and *Max* as the input features of AdaBoost, we developed a new method for predicting transcriptional enhancers: DELTA or a Distal Enhancer Locating Tool based on AdaBoost algorithm.

To train DELTA, we selected active and distal p300 binding sites overlapping DNase-I hypersensitive sites (DHSs) and distal (>2.5 kb) to UCSC TSSs as representative of enhancers (positive class set). The background set (negative class set) is composed of two types of genomic regions: UCSC TSSs overlapping DHSs as well as random genomic regions distal (>2.5 kb) to UCSC TSSs and p300 binding sites. We used a sliding window approach to predict enhancers along the genome with a step size of 100 bp. For each window, the shape and intensity parameters are calculated in both directions (forward and backward). A true enhancer with symmetric modification profiles will be assigned to high probabilities in both directions, while promoters and noise signals will be assigned to low probabilities in at least one direction. Thus, the lower probability in the two directions is treated as the final probability of a window.

**Fig 2:**
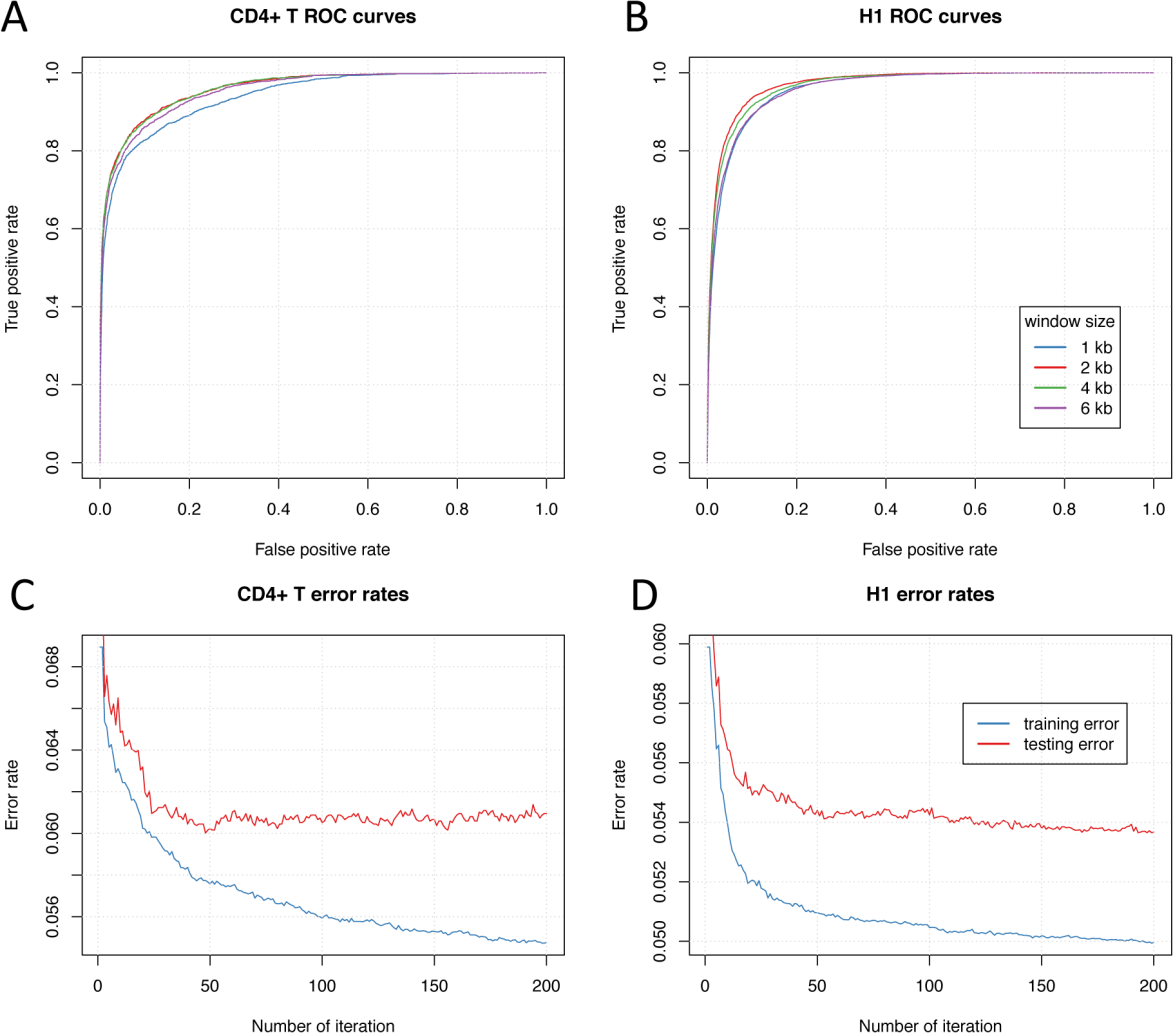
Determination of optimal window size and number of boosting iteration for DELTA. 5-fold cross-validation ROC curves at different window sizes (1, 2, 4 and 6 kb) are shown in (A) CD4^+^ T and (B) H1 cells, and a window size of 2 kb shows the largest AUC both in CD4^+^ T and H1. Train and test error curves at different numbers of iteration of AdaBoost are shown in (C) CD4^+^ T and (D) H1 cells. The train error continuously drops as number of iteration increases, but the test error becomes stable beyond 50-th iteration in CD4^+^ T and 150-th in H1. An iteration number of 150 is chosen for training AdaBoost as it appears to be optimal for both cases.

We used a 5-fold cross-validation approach and Receiver Operating Characteristic (ROC) curves to determine the optimal window size and the ratio of p300 sites to background regions in training dataset. For window size determination, we evaluated four different window sizes (1, 2, 4 and 6 kb) and found that a window size of 2 kb shows the highest Area Under the Curve (AUC) both in CD4^+^ T (AUC = 0.9606) and H1 (AUC = 0.9726), as shown in Fig 2A and 2B. To determine the ratio of positive enhancers to background regions, we compared the ROC curves of four different ratios (1:4, 1:6, 1:8 and 1:10) and found that the ROC curves of different ratios are very similar (S2 Fig). A ratio of 1:8 was used for a fair comparison with previous methods using ratios from 1:7 to 1:10 [20,21].

The number of boosting iteration is the main parameter of AdaBoost algorithm, which decides the number of decision trees. To determine the optimal number of iteration for model training, we used a 5-fold cross-validation approach to track the training and testing error rates during the whole iteration process. We found training error continuously decreased through the iteration, while testing error became stable beyond 50 iterations for CD4^+^ T and 150 for H1 cells (Fig 2C and 2D). Therefore, we chose 150 iterations for training AdaBoost as it appears to be optimal for both cases.

### Importance rankings of histone modifications are highly correlated in AdaBoost models across different cell types

We then used AdaBoost model to assess the importance of histone modifications for enhancer prediction. As an ensemble learning algorithm, AdaBoost maintains most of the desirable properties of its basic learners (decision trees in this case). A measurement of importance for predictor variables of a decision tree has been proposed previously [33]. We chose to generalize this importance measure to AdaBoost by averaging over the trees as described in [31]. Due to the stabilizing effect of averaging, AdaBoost can give a very reliable measurement of variable importance.

**Fig 3:**
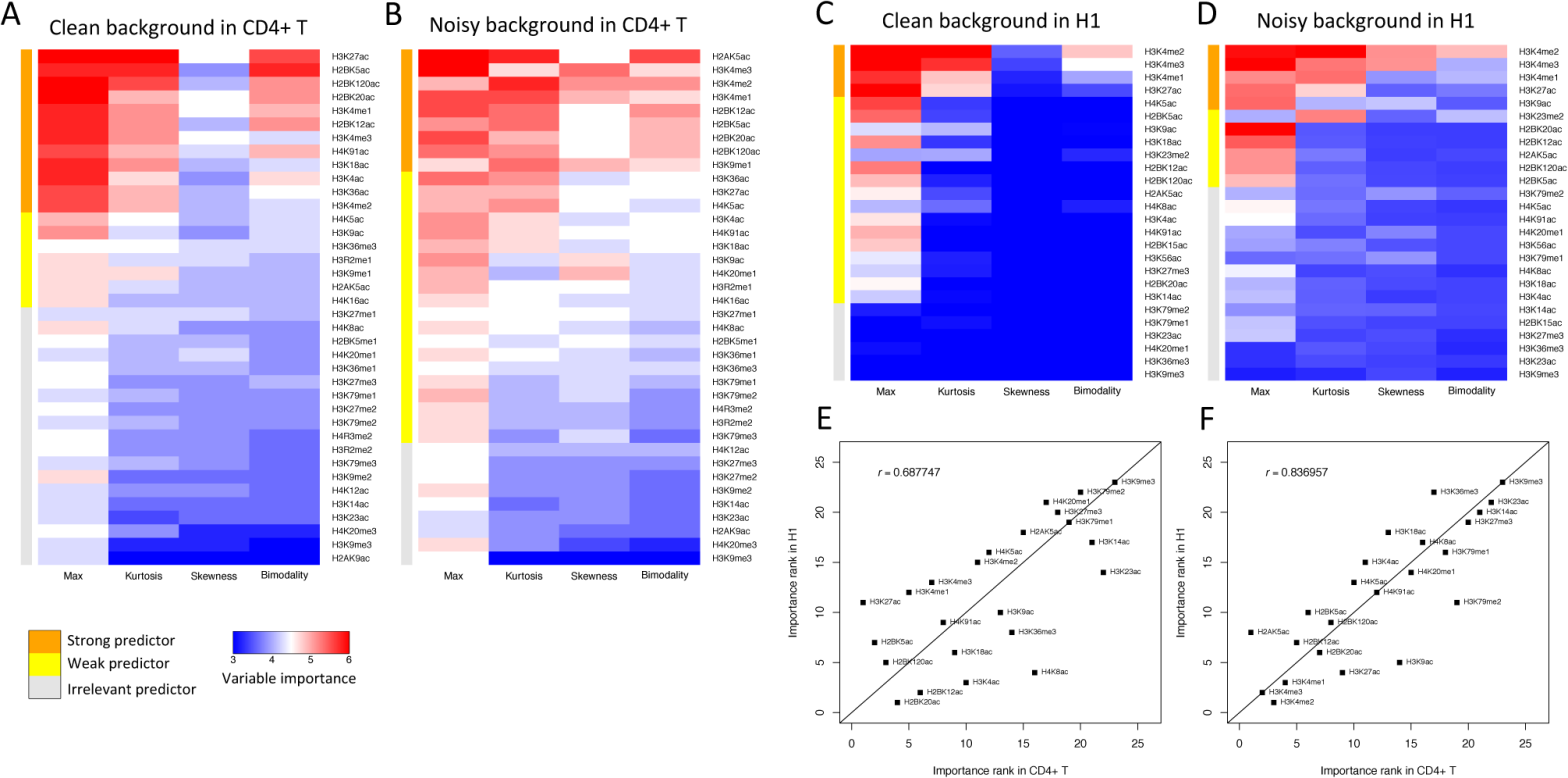
Variable importance analysis of intensity and shape parameters in CD4^+^ T and H1 cells. Histone modification clustering based on the variable importance of intensity (*Max*) and shape parameters (*Kurtosis*, *Skewness* and *Bimodality*) in CD4^+^ T and H1 cells. In CD4^+^ T, 38 histone modifications were used to train models with (A) clean background and (B) noisy background; In H1, 26 modifications were used to train models with (C) clean background and (D) noisy background. The average variable importance in 5-fold cross-validation is log transformed and represented by different colours. Clustering was performed using a K-means algorithm. Each set of modifications was classified into three groups, which were termed "Strong predictor", "Weak predictor" and "Irrelevant predictor". Scatter plot was used to show the correlation of importance ranks of histone modifications for enhancer prediction between CD4^+^ T and H1 cells. 23 shared histone modifications in models with (E) clean background and (F) noisy background were ranked by their average variable important across four parameters.

To comprehensively assess the variable importance of histone modifications for enhancer prediction, chromatin profiles of 38 and 26 histone modifications in CD4^+^ T and H1 cells were used to train the AdaBoost models, and both a clean and a noisy non-enhancer background were constructed. The clean background contains only random genomic regions distal (>2.5 kb) from UCSC TSSs and p300 binding sites, and the noisy background contains both random genomic regions and UCSC TSSs overlapping DHSs. Heatmaps (Fig 3A–3D) were used to display the variable importance of the shape and intensity parameters of histone modifications calculated by 5-fold cross-validation. When training AdaBoost model with active p300 sites and clean background, we found *Max* was the most important feature for all histone modifications both in CD4^+^ T and H1 (Fig 3A and 3C). The *Kurtosis* and *Bimodality* of several modifications also showed considerable importance, but *Skewness* didn’t exhibit any importance in both models. Interestingly, when training AdaBoost model with noisy background, we found the importance of shape parameters dramatically increased. For instance, the *Kurtosis* of H3K4me1 and H3K4me2 showed higher importance than *Max* both in CD4^+^ T and H1 (Fig 3B and 3D). The *Skewness* of promoter-enriched histone modifications (e.g., H3K4me2 and H3K4me3) showed significantly higher importance than in the case of clean background, demonstrating that *Skewness* is a specific and important shape feature for discriminating functional enhancers from noisy genome background with promoters.

To obtain an informative set of histone modifications for enhancer prediction, we used a *K*-means clustering method to automatically divide these modifications into clusters on the basis of their variable importance. An average silhouette width method was used to estimate the optimal number of clusters [34]. For both CD4^+^ T and H1, the optimal numbers of clusters were estimated to be 3. Accordingly, histone modifications in CD4^+^ T and H1 were grouped into three clusters, termed “strong predictor”, “weak predictor” and “irrelevant predictor”, respectively. Nine histone modifications in CD4^+^ T and five modifications in H1 were classified as strong predictors. Among all histone modifications, H3K4me1, H3K4me2 and H3K4me3 were the sole three modifications consistently presented in “strong predictor” sets in all four cases. Our findings as well as the similar results obtained in H1 and IMR90 by Rajagopal et al. [21] confirmed that H3K4me1, H3K4me2 and H3K4me3 were three most informative histone modifications for enhancer prediction among all available modifications.

We then investigated the relationship between the variable importance of histone modifications in CD4^+^ T and H1 cells. We ranked 23 histone modifications that presented in both datasets by their overall variable importance and examined the correlation of their rankings between two cell types. For the clean background case, we observed a correlation of 0.688 (*P*-value = 4.091E-4, Spearman’s *rho* test) between the importance rankings (Fig 3E). Because recognizing active enhancers from clean background was a relatively simple task and heavily relied on the *Max* parameter, it is easy for highly correlated modifications to provide redundant features, which will cause variability among the cell types. For the noisy background case, we observed a high correlation of 0.837 (*P*-value = 1.711E-6, Spearman’s *rho* test) between the rankings (Fig 3F), indicating a strong consistency of variable importance of histone modifications between the two distinct cell types. For comparison, we also calculated the correlation of variable importance of the noisy background using binned-vector features, which is a vector of binned reads in a window as used by previous method [21], and found a considerably lower correlation of 0.633 (*P*-value = 0.0015, Spearman’s *rho* test). Rajagopal et al. ranked 24 shared modification between H1 and IMR90 using RFECS and obtained a correlation of 0.645 (*P*-value = 6.63E-4, Spearman’s *rho* test) with noisy background training data [21], also significantly lower than that of our method. Taken together, our findings not only demonstrate that the combined set of shape and intensity features exhibits higher consistency across different cell types than single intensity and binned-vector features, but also suggest that enhancers might share common chromatin signatures across different cell types.

### Assessment of prediction accuracy of AdaBoost with different feature sets

We next assessed the prediction accuracy of AdaBoost models with different feature sets. In the case of enhancer predictions, the definition of true negatives is not straightforward, because we can never be certain that the randomly selected TSS- and p300-distal genomic regions are all non-functional. Therefore, we determined the predictive power of model by counting the percentage of predicted enhancers that overlap markers of enhancers (validation rate) or active TSSs (misclassification rate) as in the previous studies [20,21]. In addition, an F-score was computed from validation rate and misclassification rate to measure the overall performance of a prediction (see Materials and Methods). Since model training and enhancer prediction using large histone modification sets and binned-vector features is very time-consuming, we assessed prediction accuracy using randomly sampled regions from ~10% of human genome instead of the whole genome.

**Fig 4:**
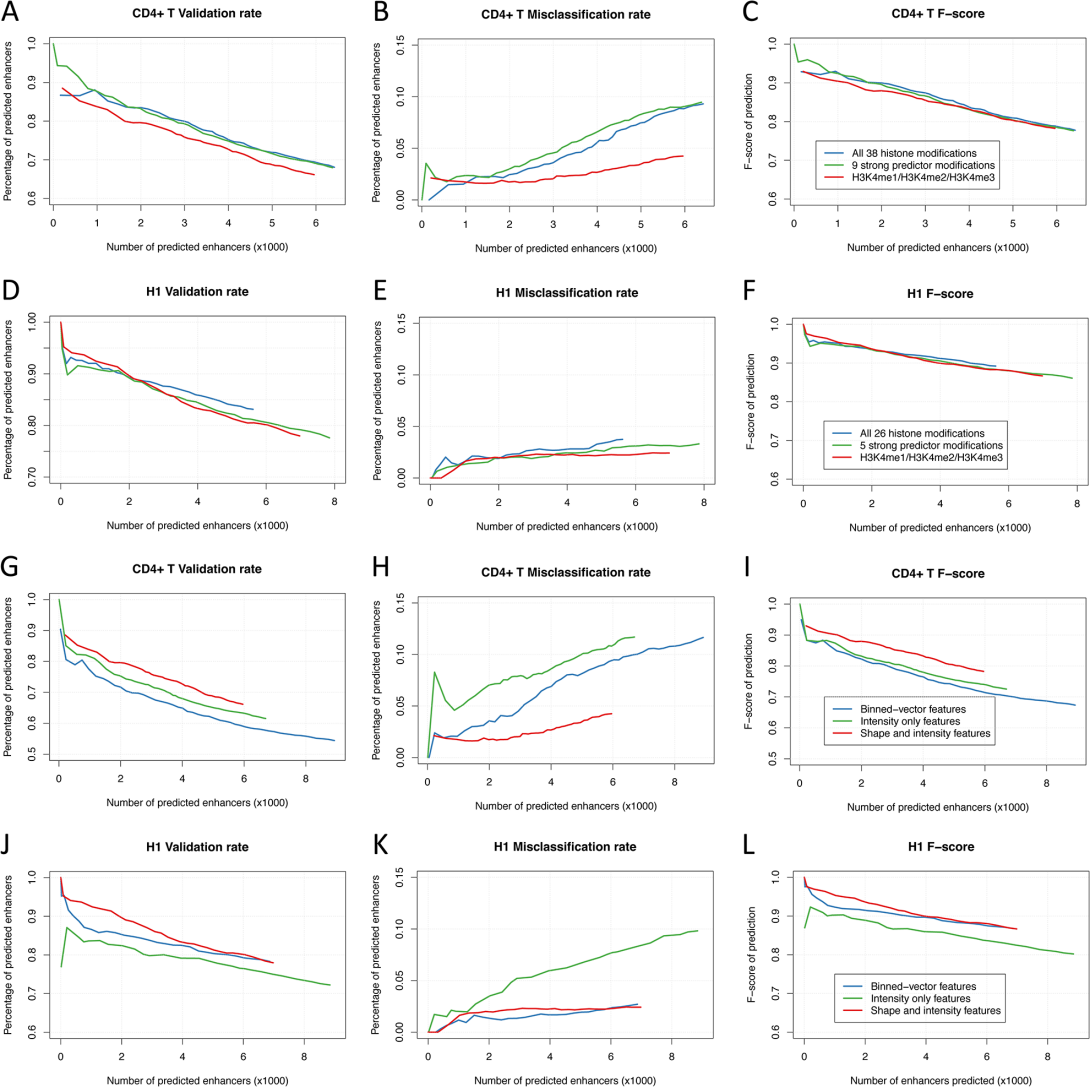
Prediction accuracy of AdaBoost models with different feature sets. (A) Validation rates, (B) Misclassification rates and (C) F-scores of enhancer predictions using AdaBoost models with three histone modification sets (all modifications, strong predictor modifications and H3K4me1/2/3) in 10% genome in CD4^+^ T and H1 (D-F). (G) Validation rates, (H) Misclassification rates and (I) F-scores of predictions using AdaBoost models with three feature extraction methods (binned-vector, intensity only and shape and intensity features) in 10% genome in CD4^+^ T and H1 (J-L). In CD4^+^ T, validation rates were measured as overlap with either p300 binding sites, DNase-I hypersensitive sites (DHS) or TF binding sites including CBP, ETS1, FOXP3, RUNX1 and STAT5, and misclassification rates were measured as overlap with UCSC TSSs, versus total number of enhancers determined by taking different probability cutoffs. In H1, validation rates were measured as overlap with either p300 binding sites, DHSs or sequence-specific TF binding sites from FactorBook, and misclassification rates were measured as overlap with UCSC TSSs, versus total number of enhancers determined by taking different probability cutoffs.

We first investigated the predictive power of different combinations of histone modifications using AdaBoost model in CD4^+^ T and H1 cells. We applied AdaBoost models with three different sets of histone modifications: all histone modifications in the datasets (38 for CD4^+^ T, 26 for H1), modifications classified as strong predictors (9 for CD4^+^ T, 5 for H1), and three core modifications H3K4me1/H3K4me2/H3K4me3 and computed validation and misclassification rate and F-score at different numbers of enhancers determined by taking different probability cutoffs. As shown in Fig 4A, 4B, 4D and 4E, we found that the all-modification set and strong-predictor set made predictions with higher validation rates but also higher misclassification rates than the H3K4me1/2/3 set. As a whole, the three sets showed a very close resemblance in F-score curves both in CD4^+^ T and H1 (Fig 4C and 4F), indicating the strong-predictor set and H3K4me1/2/3 set preserve most of the information for enhancer prediction.

We next investigated the impact of different feature extraction procedures on the predictive power of AdaBoost models. We considered three types of features: binned-vector features, intensity features and the combination of shape and intensity features, among which the former two were frequently used in previous studies [18,20,21]. We applied AdaBoost models with the three feature sets to the datasets of H3K4me1/2/3 in CD4^+^ T and H1 cells and computed validation and misclassification rate and F-score at different numbers of enhancers. We found the shape and intensity feature set performed significantly better than the intensity feature set both in CD4^+^ T and H1 (Fig 4G–4L). However, the performances of binned-vector feature set in two datasets are inconsistent. In H1, the binned-vector feature set performed as well as the shape and intensity feature set, while in CD4^+^ T it performed even worse than the intensity feature set, suggesting the high-dimensional feature vector produced by binned-vector method may cause model instability.

### Comparison of DELTA with other enhancer prediction models

**Fig 5:**
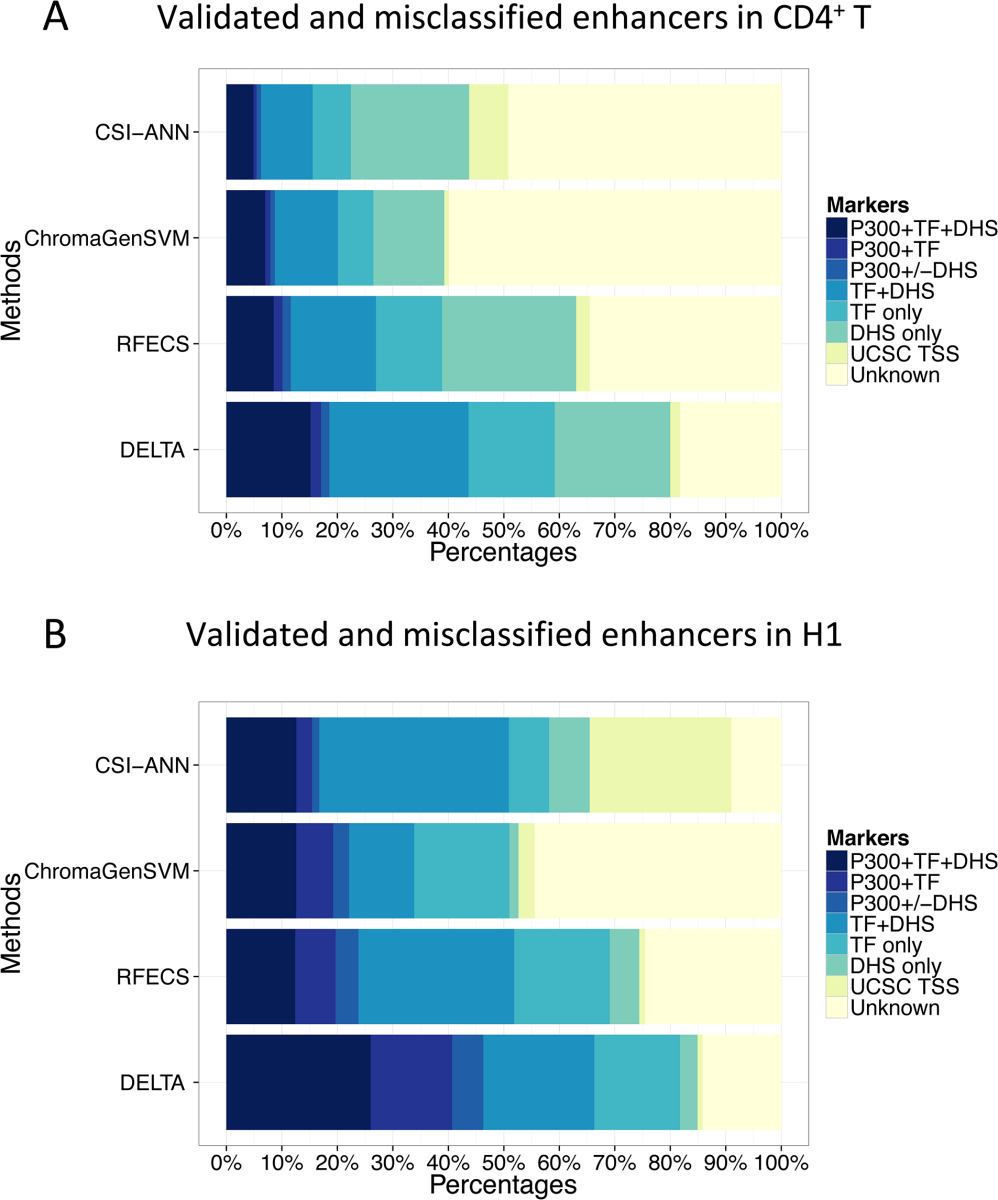
Comparison of DELTA with other enhancer prediction models. (A) In CD4^+^ T, validation rates were measured as overlap with either p300 binding sites, DNase-I hypersensitive sites (DHS) or TF binding sites including CBP, ETS1, FOXP3, RUNX1 and STAT5, and misclassification rates were measured as overlap with UCSC TSSs. (B) In H1, validation rates were measured as overlap with either p300 binding sites, DHSs or sequence-specific TF binding sites from FactorBook, and misclassification rates were measured as overlap with UCSC TSSs.

We next compared the performance of DELTA with three currently available methods for enhancer prediction: CSI-ANN, ChromaGenSVM and RFECS. The three existing methods have been applied on histone modification datasets of CD4^+^ T and H1 cells in previous studies [18,20,21]. Using histone modifications H3K4me1, H3K4me3 and H3K27ac in CD4^+^ T cells, CSI-ANN made 21832 predictions [18], ChromaGenSVM made 23574 predictions [20] and RFECS made 22947 predictions [21]. To make a fair comparison across methods, we made enhancer predictions genome-wide using DELTA with the same modifications in CD4^+^ T, and cutoff was selected that yielded a similar number of predictions (22182), as listed in S3 Table. Validation rates were computed by comparing predicted enhancers to TSS-distal p300 binding sites, DNase-I hypersensitive sites and 5 sequence-specific TFs (CBP, ETS1, FOXP3, RUNX1 and STAT5) binding sites, and misclassification rates were computed by comparing to known UCSC TSSs (see Materials and Methods). As shown in Fig 5A, we found that the validation rate of DELTA predictions is 80%, considerably higher than the other three methods (CSI-ANN 43%, ChromaGenSVM 39% and RFECS 63%), and that more than 15% DELTA predictions were supported by all three markers, also much higher than the other methods (CSI-ANN 5%, ChromaGenSVM 7% and RFECS 8%). Moreover, the misclassification rate of DELTA is 2%, considerably lower than the 7% and 3% rates of CSI-ANN and RFECS.

In H1, histone modifications H3K4me1, H3K4me2 and H3K4me3 were used for enhancer prediction. CSI-ANN made 30996 predictions [18] and ChromaGenSVM made 29598 predictions [20]. Cutoffs were selected to make a similar number of predictions for both RFECS (29500) and DELTA (30734), as listed in S4 Table. We used TSS-distal p300 binding sites, DNase-I hypersensitive sites and sequence-specific TFs binding sites to compute the validation rates. We found that more than 85% DELTA predictions were validated by at least one marker, substantially higher than the other three methods (CSI-ANN 65%, ChromaGenSVM 52% and RFECS 74%), as shown in Fig 5B. In addition, over 26% DELTA predictions were supported by all three markers, much higher than the other methods (CSI-ANN 13%, ChromaGenSVM 12% and RFECS 13%). Furthermore, the misclassification rate of DELTA is 1.6%, considerably lower than the 25%, 3.5% and 1.9% rates of CSI-ANN, ChromaGenSVM and RFECS. Overall, these results demonstrated DELTA significantly improved the prediction accuracy over current enhancer prediction techniques.

### Enhancers can be accurately predicted in one cell type by DELTA model trained on other cell types

Because the importance rankings of histone modifications in AdaBoost models are highly correlated across different cell types, we anticipated that DELTA could accurately predict enhancers in one cell type using models trained on other cell types. To test this hypothesis, the dataset of H3K4me1, H3K4me2 and H3K4me3 of five human cell types: H1, GM12878, HeLaS3, K562 and HepG2 was downloaded from the ENCODE project for model training and enhancer prediction. We also downloaded the corresponding p300 binding sites, sequence-specific TFs binding sites and DHS regions to validate the predictions. In order to validate prediction in a uniform criterion across cell types, CD4^+^ T was excluded from this analysis because the dataset of sequence-specific TFs binding sites is not available for this cell type.

**Fig 6:**
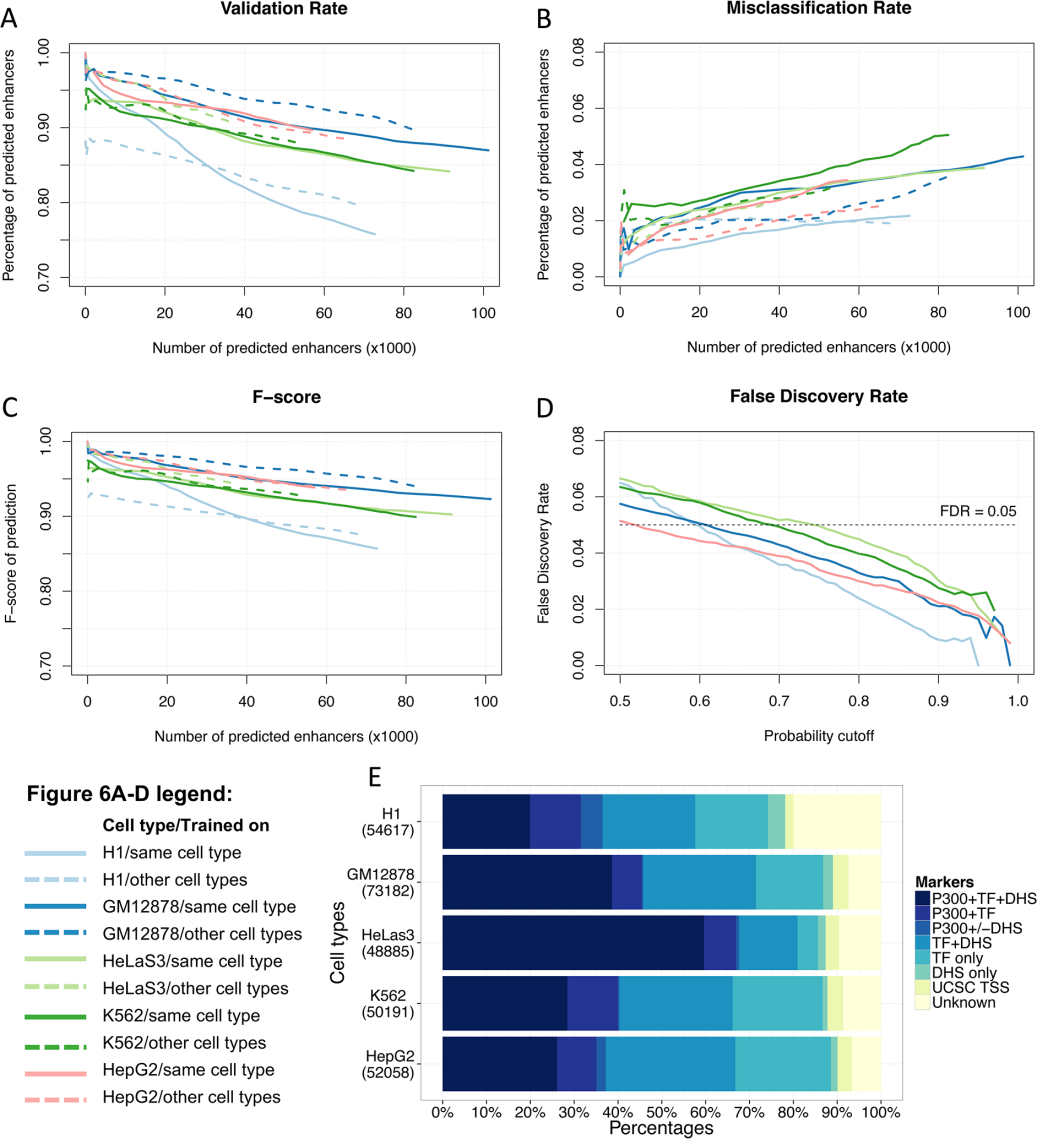
Enhancer predictions in five ENCODE cell types using DELTA. (A) Validation rates, (B) Misclassification rates and (C) F-scores of enhancer predictions using AdaBoost models trained in the same cell type (solid lines) and other four cell types (dashed lines) in five cell types. Validation rates were measured as overlap with either p300 binding sites, DNase-I hypersensitive sites (DHS) or sequence-specific TF binding sites from FactorBook, and misclassification rates were measured as overlap with UCSC TSSs, versus total number of enhancers determined by taking different probability cutoffs. (D) False Discovery Rate (FDR) for each cell type plotted as a function of prediction probability. (E) Percentages of validated and misclassified enhancer predictions in five cell types at a FDR of 5% with number of enhancer predictions shown at the left of the bar.

Enhancers were predicted in each of the five ENCODE cell types using two AdaBoost models: one trained on the same cell type and the other trained on a combined dataset of the other four cell types. For the combined dataset, 1000 representative enhancers (active and distal p300 binding sites) and 8000 background regions (4000 active TSSs and 4000 random and distal genomic regions) were randomly sampled from each of the four cell types and then constituted a “multicell” training set. Validation rate, misclassification rate and F-score were calculated at each cutoff as described previously to evaluate prediction accuracy. We compared the predictions made by the two AdaBoost models and found that the two prediction sets exhibited very similar validation rate, misclassification rate and F-score curves in all five cell types, as shown in Fig 6A–6C, indicating that DELTA can accurately predict enhancers in one cell type using models trained on independent cell types without loss of prediction accuracy. We also carried out the comparisons between the two models as described above using binned-vector parameters in the five cell types and found a decrease in F-score when predicting enhancers using combined datasets of other cell types than using the dataset of the same cell type (S3 Fig).

A set of high-confidence enhancer predictions in multiple cell types would contribute substantially to the functional annotation of human non-coding genomic regions and to the reliable construction of genetic regulatory networks (GRNs). We predicted enhancers in the five ENCODE cell types using DELTA models trained on the same cell type. When predicting enhancers across multiple cell types, it is preferable to have enhancer predictions with the same level of confidence. We calculate a False Discovery Rate (FDR) to determine the appropriate cutoff for each cell type. Two cases are treated as false discovery evens: declaration as enhancer at permuted genomic regions and declaration as enhancer at active TSSs. The ratio of false discovery evens/enhancers predicted in real data is computed as FDR at various cutoffs (Fig 6D). The contributions of the two components to FDR in five cell types are shown in S4 Fig. Using an FDR of 5%, we predicted a consistent set of high-confidence enhancers in the five ENCODE cell types, as listed in S5 Table. The numbers of enhancer predictions in five cell types range from 48885 to 73182 as shown at the left of the bar in Fig 6E. The validation rates are above 85% for all cell types except for H1 and the misclassification rates are all below 5%. Besides, 20–60% of the predicted enhancers are marked by all three types of experimental evidences. We examined the correlation between the numbers of available TFs in five cell types and the corresponding validation rates and didn’t find a significant correlation between numbers of TFs and validation rates (*P*-value = 0.35, Spearman’s rank correlation test).

We next compared these predictions with those of ChromHMM in four shared cell types. We found the numbers of enhancers predicted by ChromHMM (H1: 619061, GM12878: 571339, HepG2: 546343 and K562: 622257) are one magnitude order larger than those predicted by DELTA with a FDR of 0.05. Despite the great difference in the number of predictions, we found the vast majority of the predictions by DELTA overlapped with Weak/Strong enhancers states of ChromHMM (H1: 98.8%, GM12878: 99.0%, HepG2: 98.5% and 95.9%). Moreover, we examined the validation rates and misclassification rates of ChromHMM in the four cell types and found it can achieve validation rates of 60.5–69.6% and misclassification rates of 9.4–10.5%, as shown in S5 Fig.

In addition, we found enhancers predicted by both the same-cell type model and cross-cell type model are highly cell-type specific. As shown in S6 Fig., we randomly sampled 10000 predicted enhancers from each cell type and found ~86–92% predicted enhancers are specific to one cell type. Very few enhancers are shared by three or more cell types simultaneously and only ~2–3% enhancers are shared by two different cell types. This ratio is lower than the observation (~6%) in a previous study in HeLas3 and K562 using only p300-positive enhancers [35].

In summary, we developed a method for accurately predicting enhancers across different cell types and obtained a high-confidence set of enhancer predictions in multiple ENCODE cell lines with the same level of confidence. This will enable us to systemically locate and annotate the regulatory non-coding genomic regions in a wide range of human cell lines.

Discussion In recent years, the chromatin modification data of a growing number of cell types have been obtained with the progress of ENCODE and Roadmap epigenomics projects, but accurately predicting DNA regulatory elements from these chromatin data remains a great challenge, as current prediction tools are limited in their prediction accuracy and ability to reliably predict regulatory elements in new cell types. In this paper, we described a novel method for predicting transcriptional enhancers on the basis of shape features of histone modifications and AdaBoost algorithm. There are several advantages of our method that will significantly contribute to the field of enhancer prediction.

Initially, we developed an efficient dimension reduction method by systemically introducing and assessing a set of shape parameters of histone modifications profiles. We showed that these parameters can accurately reflect the distributional characteristics of histone modifications at promoters and enhancers and that the combined shape and intensity parameters could achieve significantly higher prediction accuracy than the previously used feature sets (intensity features and binned-vector features), indicating it is a more advanced method for feature extraction of chromatin modification profiles than the current techniques.

In addition, we developed a new automatic approach for selecting informative histone modifications for enhancer prediction from a variety of modifications. AdaBoost models with small decision trees also gave information on the variable importance of histone modifications for making enhancer predictions. We used an unsupervised clustering method to automatically classify histone modifications into different groups according to their variable importance for enhancer prediction. We found that predictions made by AdaBoost using modifications termed as “strong predictor” closely resembled those made by the full set of modifications in prediction accuracy. We also confirmed that H3K4me1/2/3 constitute a robust modification set that keeps most of the information for enhancer prediction in different datasets. These findings indicate combining AdaBoost algorithm with automatic clustering method is an efficient way to select importance variables from a variety of histone modifications.

Furthermore, our enhancer prediction method significantly outperformed existing methods in prediction accuracy. Three experimental markers of enhancers, including p300 binding sites, sequence-specific TF binding sites and Dnase-I hypersensitive sites, were used to evaluate the prediction accuracy. We found DELTA improves the validation rates of at least one marker on the previous methods RFECS, CSI-ANN and ChromaGenSVM by 13.3–105% relatively and the validation rates of three markers by 87.5–300% relatively in CD4^+^ T and H1 cells.

Moreover, DELTA is the first model that successfully predicts enhancers in one cell type using models trained in other cell types without loss of accuracy. We attribute this success to the non-redundant shape parameters of histone modification that are highly consistent across different cell types, because the AdaBoost models with binned-vector features didn’t show such property. This finding also indicates a general mechanism of histone modifications acting on transcriptional enhancers.

In summary, all above features make DELTA a powerful and reliable tool for predicting enhancers in a variety of cell types. We also want to mention that the entire workflow of this study, including shape feature extraction, variable importance assessment and model training and prediction, can be conveniently applied to identify other types of DNA elements with distinct chromatin signatures.

## Materials and Methods Data Source

For CD4^+^ T cells, the ChIP-seq data of 38 histone modifications were obtained from Wang *et al*. [36]. The p300 ChIP-seq data was obtained from Wang *et al*. [37], the DNase-I data was obtained from Boyle *et al*. [38] and the ChIP-seq data of five sequence-specific transcription factors: CBP, ETS1, FOXP3, RUNX1 and STAT5 were obtained from Schmidl *et al*. [39]. For H1 cells, the data of 26 histone modifications was downloaded from NIH Roadmap Epigenome Project page (http://www.genboree.org/EdaccData/) [40] and the p300 ChIP-seq data was downloaded from NCBI GEO under accession number GSE37858 [21]. For cell lines GM12878, HeLas3, K562 and HepG2, the ChIP-seq data of three histone modifications H3K4me1, H3K4me2 and H3K4me3 were downloaded from the UCSC ENCODE [41] under the GEO accession number GSE29611, and p300 ChIP-seq data was downloaded from UCSC ENCODE page (http://hgdownload.cse.ucsc.edu/ goldenPath/hg19/encodeDCC/wgEncodeSydhTfbs/). We obtained sequence-specific TFs binding sites from FactorBook Version 3 dataset at UCSC ENCODE website (http://hgdownload.cse.ucsc.edu/goldenpath/hg19/encodeDCC/wgEncodeRegTfbsClustered/). The numbers of available TFs in H1, HeLas3, HepG2, GM12878 and K562 are 28, 30, 40, 47 and 50 respectively. [42]. Narrow DNase-I peaks of the five cell lines were downloaded from ENCODE page (http://hgdownload.cse.ucsc.edu/goldenPath/hg19/encodeDCC/wgEncodeUwDnase/). Any data mapped to hg18 or hg17 genome version was converted to hg19 using UCSC liftover tool [43].

### Data normalization and smoothing

To obtain histone modification profiles, the ChIP-seq reads were first binned into short intervals (100 bp). The number of reads in each interval was normalized against the total read count by using an RPKM-like measure [44]. In order to lower data noise, the Hanning window function [45] was employed to smooth the profiles of histone modifications by correcting the RPKM value of each bin based on the RPKM values of its neighbourhoods.

### Peak calling

To determine the binding sites of p300 and other five TFs: CBP, ETS1, FOXP3, RUNX1 and STAT5, we used MACS [46] software to call peaks from their ChIP-seq data. ChIP-seq input files were used as background and default p-value cutoff of 1e-5 was used.

### Defining the shape features of histone modification ChIP-seq signal

To describe the shape features of a ChIP-seq signal, we employed three descriptive parameters defining three distinct aspects of a probability distribution. Their definitions are listed as follows:

*Kurtosis*: *Kurtosis* measures the “sharpness” of a probability distribution, ranging from 1 for uniform distribution to infinity for extremely sharp distribution. It can be quantified in different ways, and one common measure is based on a scaled version of the fourth moment, which is defined as:

$$\text{Kurt}=\frac{\text{E}[{\left(X-\mu \right)}^{4}]}{{\left(\text{E}{\left[X-\mu \right]}^{2}\right)}^{2}}-3=\frac{\mu_{4}}{\sigma^{4}}-3$$ (1)

In this study, we used a sample *Kurtosis* to quantify the sharpness of a ChIP-seq signal. Let *x*_*i*_ index the relatively position of the *i*-th read in a sliding window, $\bar{x}$ index the average position of all *n* reads in the window. The *Kurtosis* of the ChIP-seq signal in this window can be calculated as follows:

$$\text{kurt}=\frac{m_{4}}{m_{2}^{2}}-3=\frac{\frac{1}{n}\sum_{i=1}^{n}{\left(x_{i}-\bar{x}\right)}^{4}}{{\left(\frac{1}{n}\sum_{i=1}^{n}{\left(x_{i}-\bar{x}\right)}^{2}\right)}^{2}}-3$$ (2)

*Skewness*: *Skewness* measures the asymmetry of the probability distribution of a random variable about its mean, ranging from -1 for extremely right-skewed distribution to +1 for extremely left-skewed distribution, and a zero value indicates symmetric distribution. The population *Skewness* can be defined as:

$$\text{Skew}=\frac{\mu_{3}}{\sigma^{3}}=\frac{\text{E}[{\left(X-\mu \right)}^{3}]}{{\left(\text{E}{\left[X-\mu \right]}^{2}\right)}^{3/2}}$$ (3)

We used a sample *Skewness* as follows to quantify the extent of a ChIP-seq signal “leans” to one side of the mean:

$$\text{skew}=\frac{m_{3}}{m_{2}^{3/2}}=\frac{\frac{1}{n}\sum_{i=1}^{n}{\left(x_{i}-\bar{x}\right)}^{3}}{{\left(\frac{1}{n}\sum_{i=1}^{n}{\left(x_{i}-\bar{x}\right)}^{2}\right)}^{3/2}}$$ (4)

*Bimodality*: *Bimodality* is a describer to indicate a probability distribution is bimodal or unimodal. Here we use Sarle’s bimodality coefficient to represent the *Bimodality*, which is defined as:

$$\text{BI}=\frac{{\text{Skew}}^{2}+1}{\text{Kurt}}$$ (5)

*Bimodality* ranges from 0 to 1, and value greater than 5/9 indicate a bimodal distribution. We used the following equation to calculate the *Bimodality* of the ChIP-seq signal in a window:

$$\text{bi}=\frac{{\text{skew}}^{2}+1}{\text{kurt}+\frac{3{\left(n-1\right)}^{2}}{\left(n-2)(n-3\right)}}$$ (6)

The intensity of a ChIP-seq signal is a feature frequently used in previous enhancer prediction methods. We used a *Max* parameter to measure the abundance of histone modification. It is defined as the maximum RPKM value of bins in a window.

### Training dataset

In order to train AdaBoost models, p300 binding sites overlapping with DNase-I hypersensitive sites and distal to annotated TSS were used as representative of positive enhancers (true positives). For negative training data, we constructed both a clean and a noisy non-enhancer background. The clean background only contains random genomic regions distal from known TSSs and enhancers, while the noisy background contains both random genomic regions and annotated TSSs overlapping with DNase-I locations. The clean background was only used in the variable importance assessment procedure.

### Prediction validation

In the case of enhancer predictions, the definition of true negatives is not straightforward, because we can never be certain that the randomly selected TSS- and p300-distal genomic regions are all non-functional. Therefore, to compare model performances, we used three types of experimental markers of enhancers, including p300 binding sites (excluding those used in training), a wide range of sequence-specific transcription factors binding sites (TFBSs) obtained from FactorBook (http://www.factorbook.org/) [42] and other studies [37,39], and DNase-I hypersensitive sites (DHSs) downloaded from ENCODE (http://genome.ucsc.edu/ENCODE/) [41]. The binding sites of CTCF were removed from the validation set since it is known as a marker of insulators [47]. We recorded the percentages of predicted enhancers that overlap these markers as validation rates and the percentages of predicted enhancers that overlap DHS-positive UCSC TSSs [43] as misclassification rates. Specifically, predicted enhancers are classified as ‘‘validated”, ‘‘misclassified” or ‘‘unknown” based on the following criteria: 1) if the nearest experimental evidence lies within 1 kb of the enhancer and the nearest TSS is greater than 2.5 kb away from the evidence, the enhancer is ‘‘validated”. A window of -1 to +1 kb is a relatively strict criterion for validation compared to those used by previous studies (-2.5 to 2.5 kb for RFECS and ChromaGenSVM, -1.25 to 1.25 kb for CSI-ANN); 2) if a TSS lies within 2.5 kb of the enhancer and the nearest experimental evidence is greater than 1 kb away, the enhancer is ‘‘misclassified”; 3) if there is no experimental evidence or TSS within 2.5 kb of the enhancer, it is “unknown”. In addition, we compute an F-score based on validation rate and misclassification rate to measure the overall performance of a prediction:

$$\text{F-score}=\frac{2\times \text{Validation Rate}\times (1-\text{Misclassification Rate})}{\text{Validation Rate}+(1-\text{Misclassification Rate})}$$ (7)

### Software implementation

The DELTA software was implemented in Python and R. Specifically, reads counting, FPKM normalization and profile smoothing was implemented in Python using the “pybedtools” package [48]. Shape parameter computing was implemented in Python using the “scipy” package. The AdaBoost algorithm is implemented in R using ‘ada’ package [49] and decision trees were used as the basic learner of AdaBoost during its boosting iteration procedure. The DELTA software and source code is freely available from GitHub (https://github.com/drlu/delta).

## Supporting Information

S1 Fig Boxplots of the four parameters and significances of the difference between p300 and MED1-binding sites for B) H3K4me1, C) H3K4me2 and D) H3K4me3 in ENCODE regions of HeLas3 cells. Significance (*P*-value) of the difference between two means was calculated by Wilcoxon rank-sum difference test.
(TIF)

S2 Fig 5-fold cross-validation ROC curves at different ratios of p300:background in A) CD4^+^ T and B) H1 cells. (TIF)

S3 Fig Enhancer predictions in five ENCODE cell types using DELTA with binned-vector features. A) Validation rates, B) Misclassification rates and C) F-scores of enhancer predictions using AdaBoost models trained in the same cell type (solid lines) and other four cell types (dashed lines) in five cell types. Validation rates were measured as overlap with either p300 binding sites, DNase-I hypersensitive sites (DHS) or sequence-specific TF binding sites from FactorBook, and misclassification rates were measured as overlap with UCSC TSSs, versus total number of enhancers determined by taking different probability cutoffs.
(TIF)

S4 Fig Two components of False Discovery Rate (FDR) plotted as a function of prediction probability in A) H1, B) GM12878, C) HeLas3, D) HepG2 and E) K562 cells. (TIF)

S5 Fig Evaluation of enhancers predicted by ChromHMM in four ENCODE cell types. Validation rates were measured as overlap with either p300 binding sites, DHSs or sequence-specific TF binding sites from FactorBook, and misclassification rates were measured as overlap with UCSC TSSs.
(TIF)

S6 Fig Venn diagrams of enhancers predicted by A) same-cell type and B) cross-cell type model in five ENCODE cell types. (TIF)

S1 Table The mean values of four parameters and corresponding significances (*P*-values) of the differences between enhancers and promoters of 38 histone modifications in CD4^+^ T. (XLSX)

S2 Table The mean values of four parameters and corresponding significances (*P*-values) of the differences between enhancers and promoters of 26 histone modifications in H1. (XLSX)

S3 Table Enhancer predictions (22182) in CD4^+^ T cells using DELTA with H3K4me1, H3K4me3 and H3K27ac. (XLSX)

S4 Table Enhancer predictions (30734) in H1 cells using DELTA with H3K4me1, H3K4me2 and H3K4me3. (XLSX)

S5 Table Enhancer predictions using DELTA with H3K4me1, H3K4me2 and H3K4me3 at a FDR of 5% in H1, GM12878, HeLas3, K562 and HepG2 cells (XLSX)
