# Supplementary figures and images for: DELTA: A Distal Enhancer Locating Tool Based on AdaBoost Algorithm and Shape Features of Chromatin Modifications
Source: PLoS One. 2015 Jun 19;10(6):e0130622. doi: 10.1371/journal.pone.0130622 (PMC4474808; doi:10.1371/journal.pone.0130622)

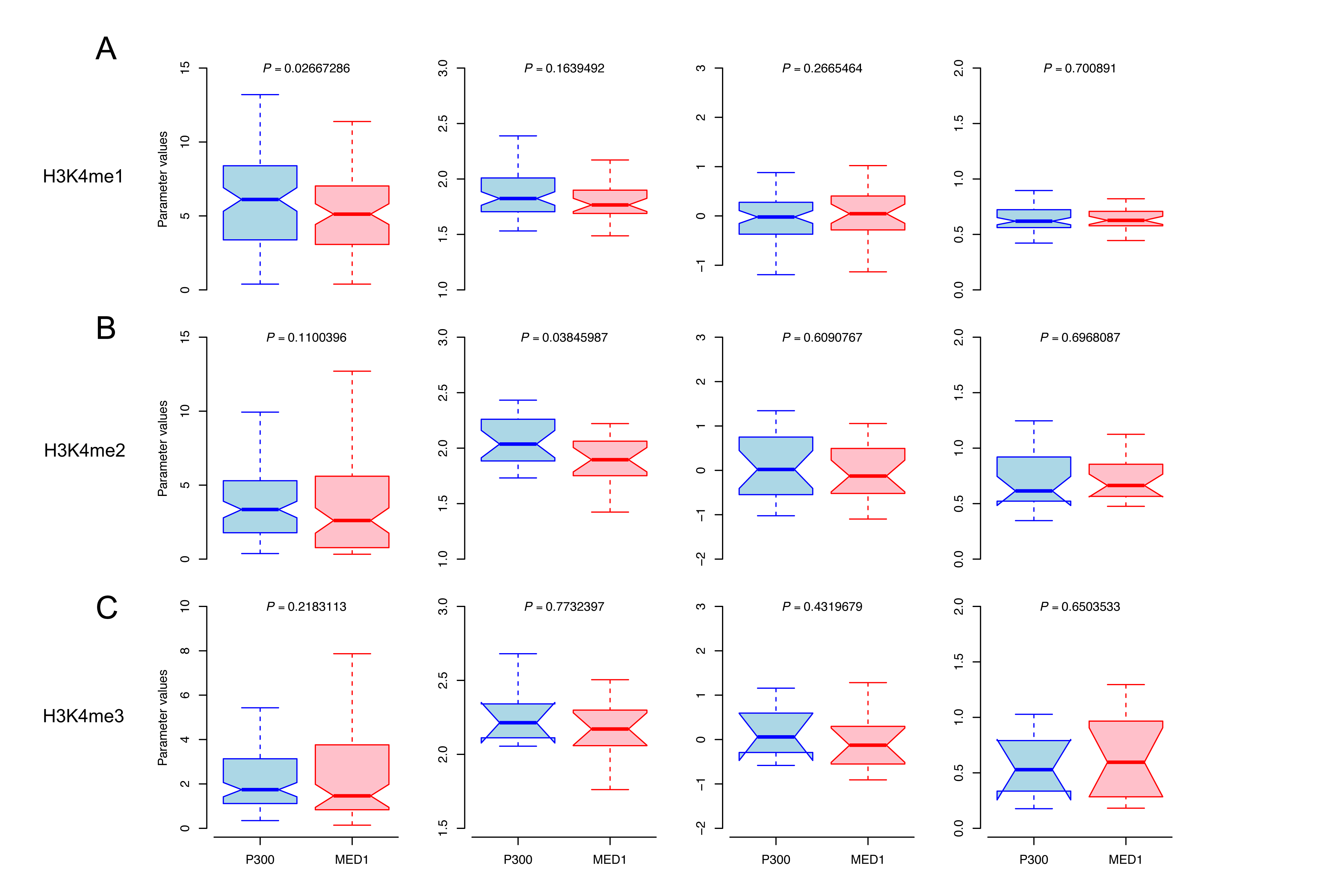

Supplement: S1 Fig — Significance (P-value) of the difference between two means was calculated by Wilcoxon rank-sum difference test. (TIF) [file pone.0130622.s001.tif]

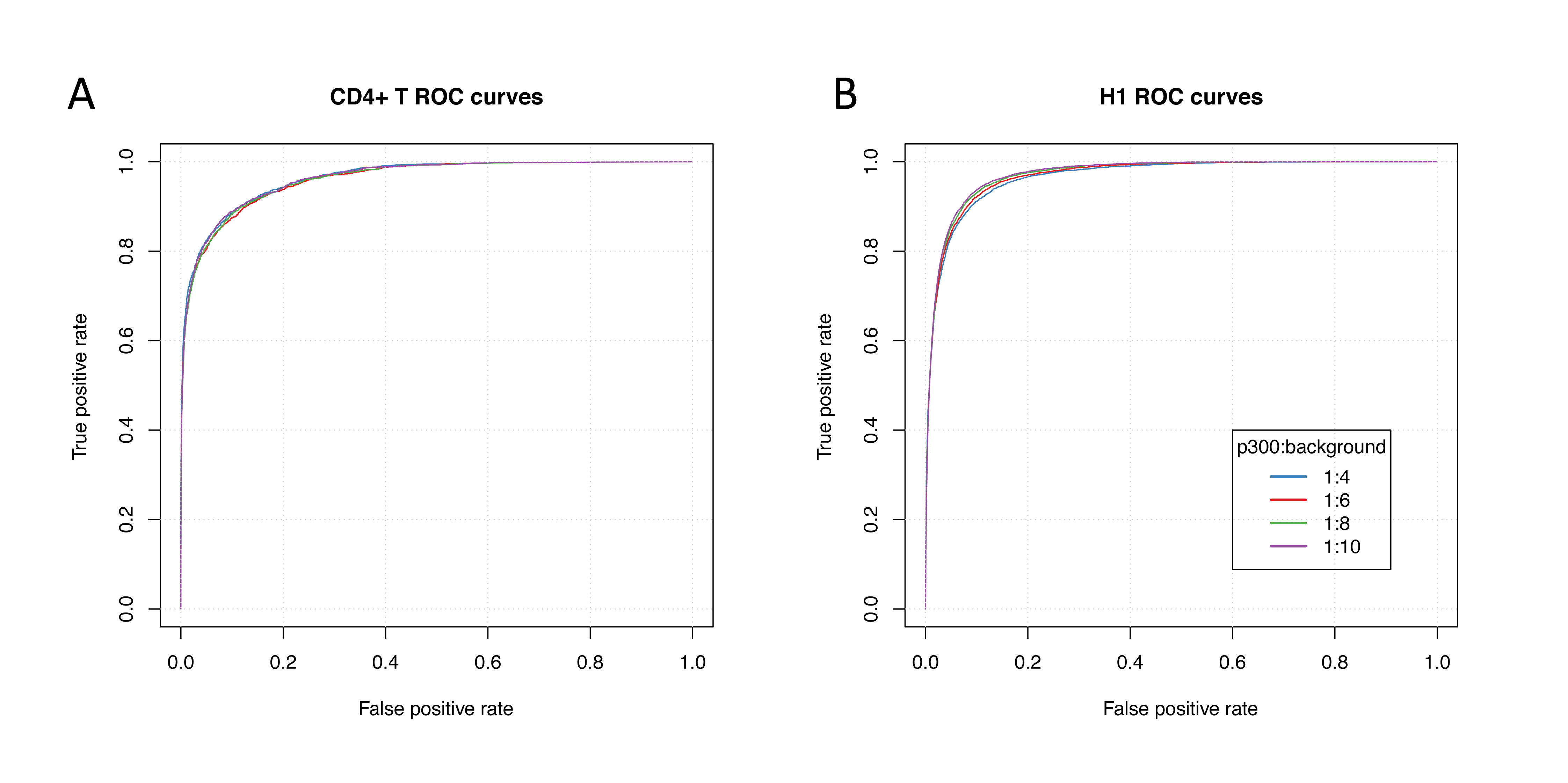

Supplement: S2 Fig — (TIF) [file pone.0130622.s002.tif]

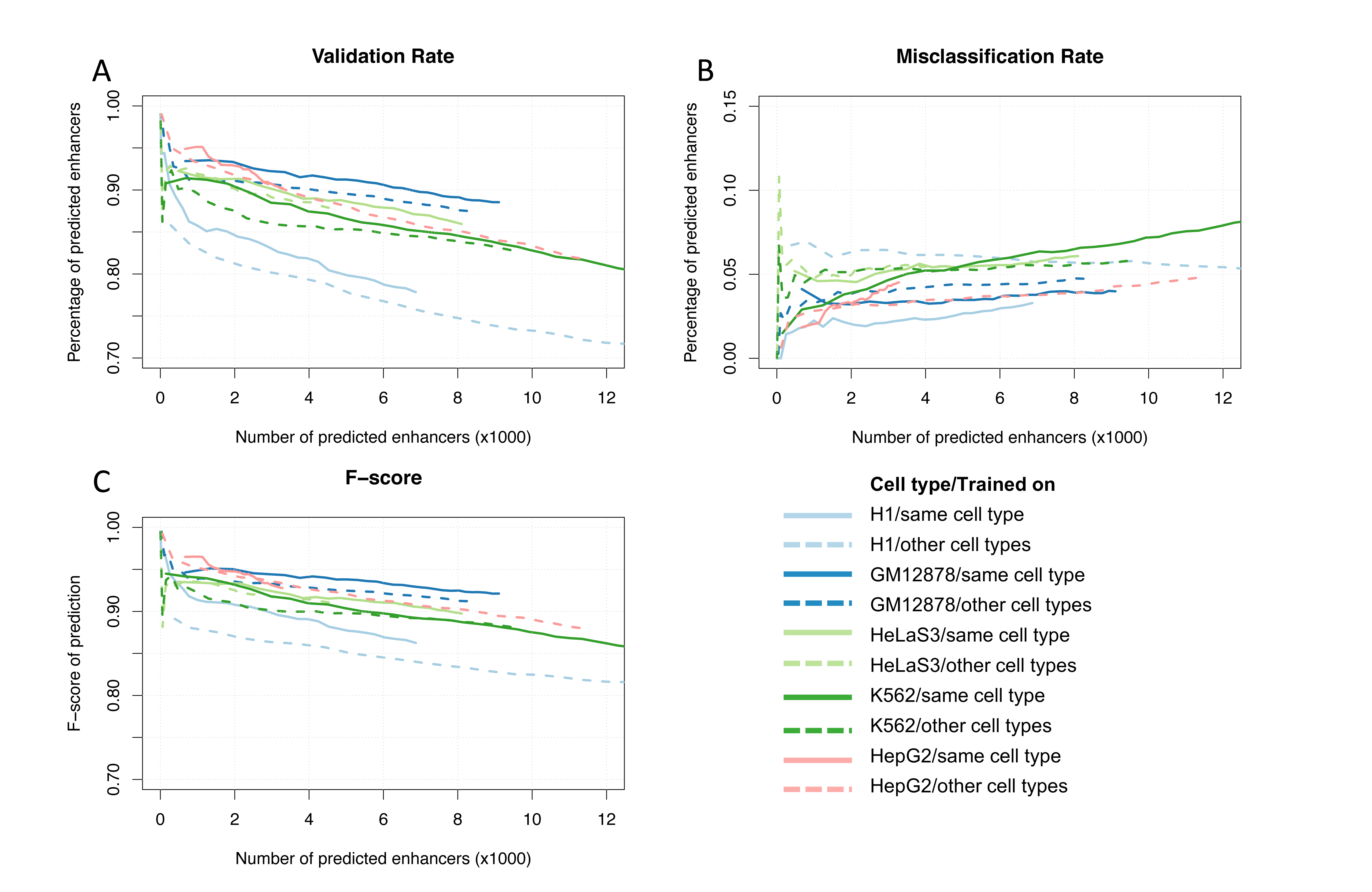

Supplement: S3 Fig — A) Validation rates, B) Misclassification rates and C) F-scores of enhancer predictions using AdaBoost models trained in the same cell type (solid lines) and other four cell types (dashed lines) in five cell types. Validation rates were measured as overlap with either p300 binding sites, DNase-I hypersensitive sites (DHS) or sequence-specific TF binding sites from FactorBook, and misclassification rates were measured as overlap with UCSC TSSs, versus total number of enhancers determined by taking different probability cutoffs. (TIF) [file pone.0130622.s003.tif]

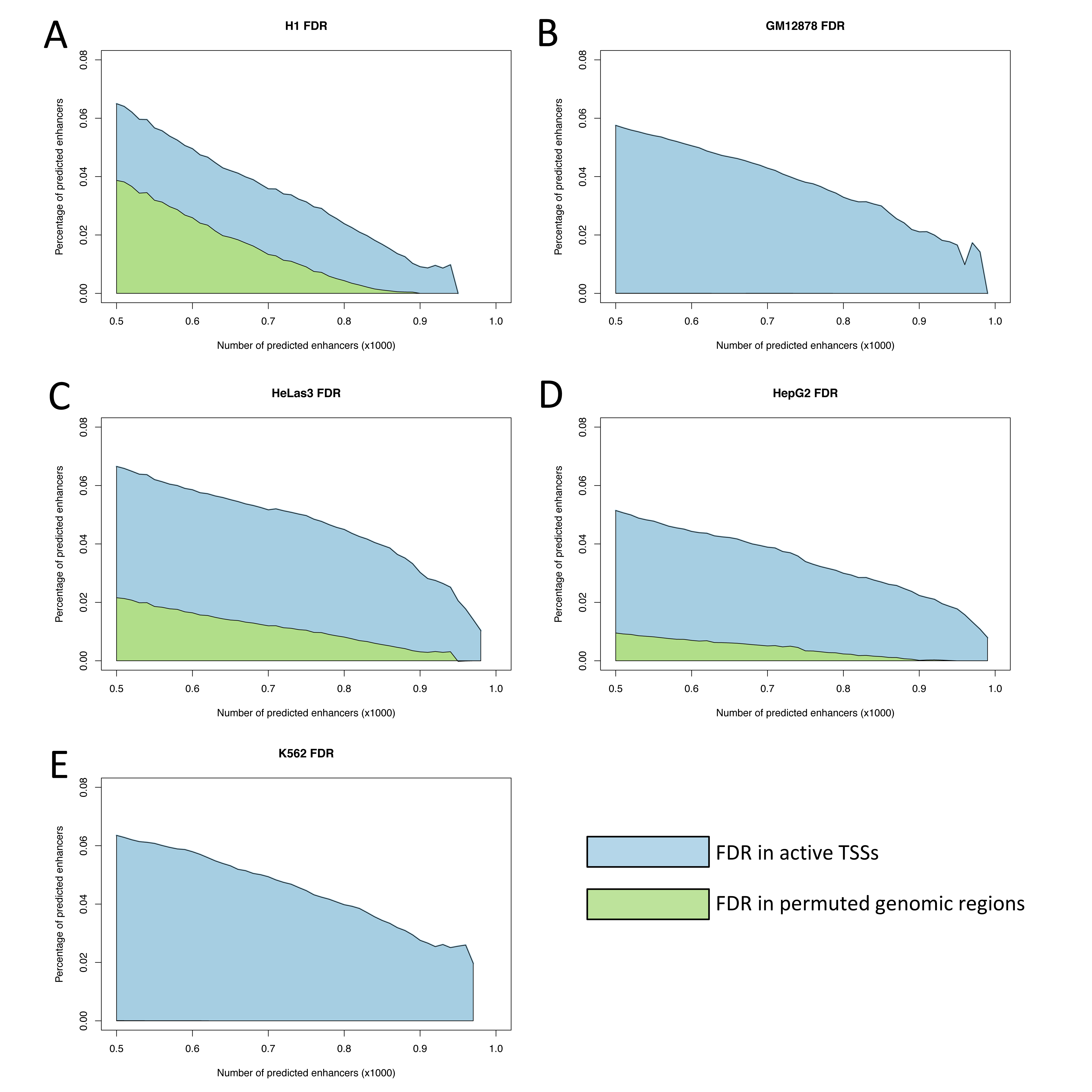

Supplement: S4 Fig — (TIF) [file pone.0130622.s004.tif]

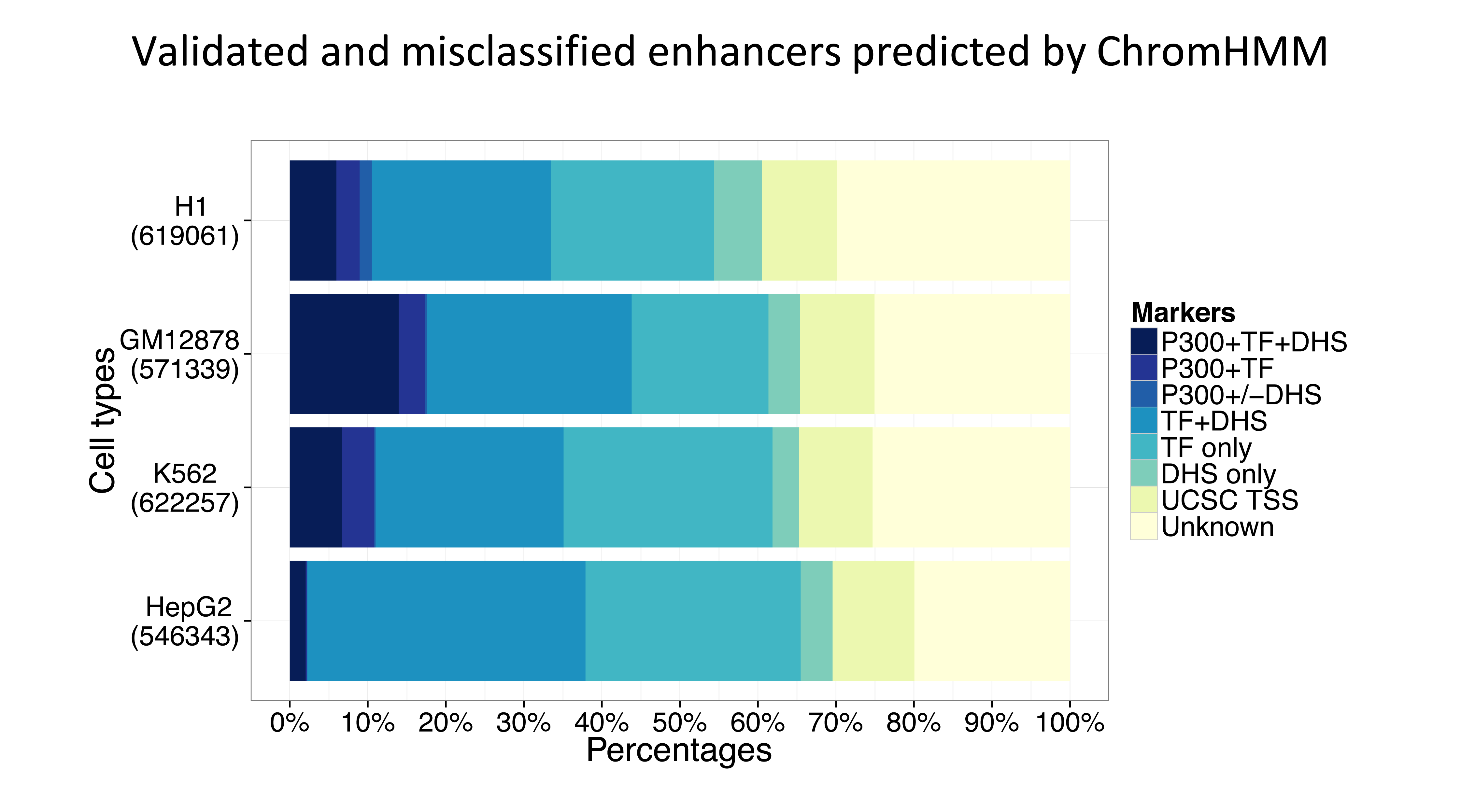

Supplement: S5 Fig — Validation rates were measured as overlap with either p300 binding sites, DHSs or sequence-specific TF binding sites from FactorBook, and misclassification rates were measured as overlap with UCSC TSSs. (TIF) [file pone.0130622.s005.tif]

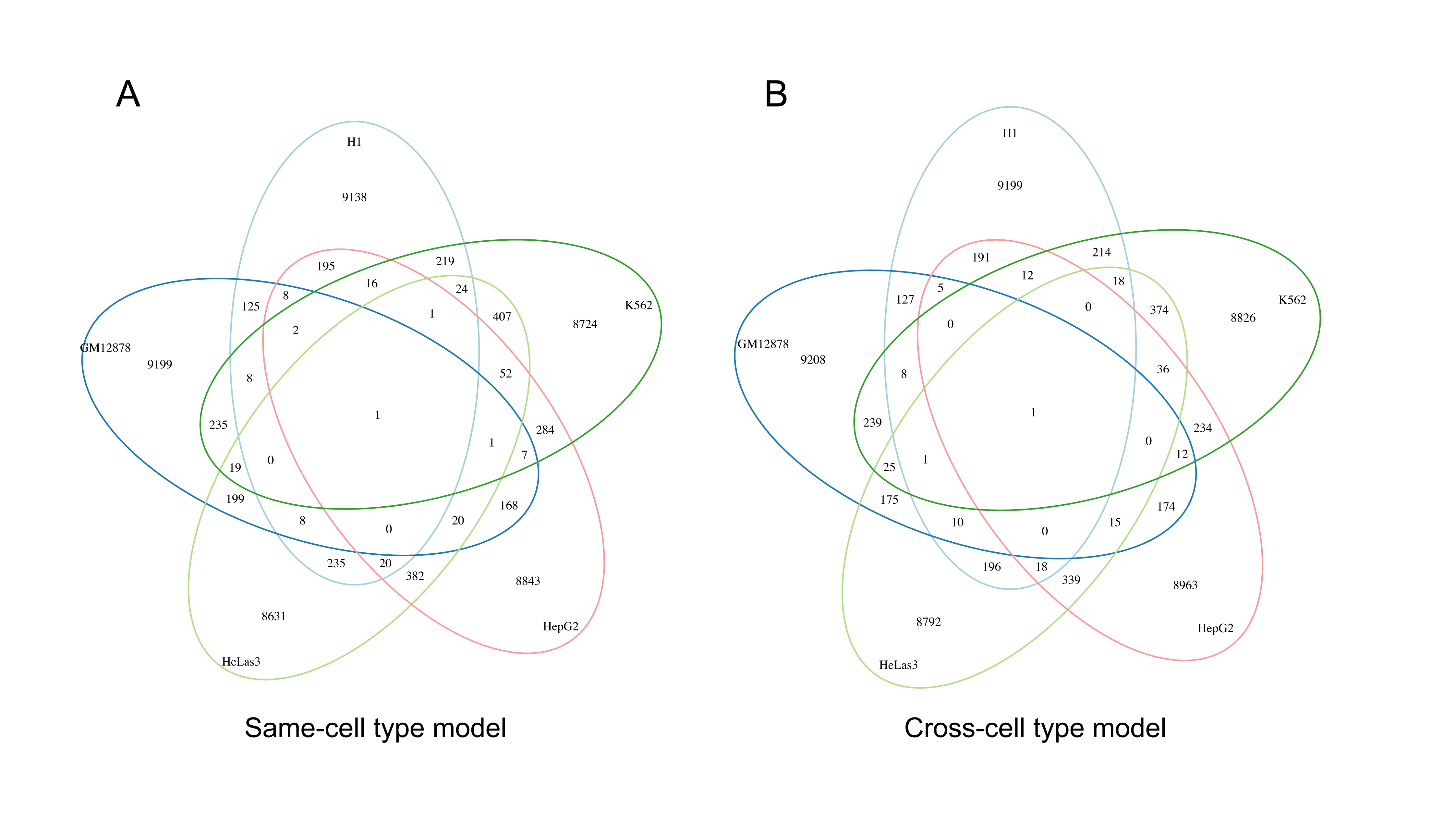

Supplement: S6 Fig — (TIF) [file pone.0130622.s006.tif]
